# SN38-PEG-PLGA-verapamil nanoparticles inhibit proliferation and downregulate drug transporter ABCG2 gene expression in colorectal cancer cells

**DOI:** 10.1007/s40204-017-0073-y

**Published:** 2017-09-25

**Authors:** Zahra Nagheh, Shiva Irani, Reza Mirfakhraie, Rassoul Dinarvand

**Affiliations:** 1 Department of Biology, Science and Research Branch, Islamic Azad University, Tehran, Iran; 2 Department of Medical Genetics, Shahid Beheshti University of Medical Sciences, Tehran, Iran; 3 Nanotechnology Research Center, Faculty of Pharmacy, Tehran University of Medical Sciences, 1417614411 Tehran, Iran; 4 Department of Pharmaceutics, Faculty of Pharmacy, Tehran University of Medical Sciences, Tehran, Iran

**Keywords:** Colon cancer, SN38, Nanoparticle, ABCG2, BAX/BCL2 expression ratio

## Abstract

Nowadays, nanoparticle-based drug delivery systems are recognized to reduce the therapeutic side effects. One of the common problems in cancer treatment is cancer drug resistance, resulting from the over-expression of one energy-dependent transporter that enhances drug efflux. Irinotecan is used for metastatic colorectal cancer. The involvement of ABCG2 transporter in irinotecan resistance has been established. The current study was designed to characterize SN38-loaded pegylated (polyethylene glycol) PLGA [poly(lactic-*co*-glycolic acid)]-verapamil nanoparticles (NPs), and to distinguish the cytotoxic effect of SN38-PEG-PLGA-Ver NPs and the ability of SN38-PEG-PLGA-Ver NPs to inhibit drug resistance through the inhibition of ABCG2 expression. The surface morphology of nanoparticles was determined by scanning electron microscopy. The drug cytotoxicity of SN38-PEG-PLGA-verapamil nanoparticles was measured by MTT assay with desired concentrations and SN38-PEG-PLGA-Ver at different incubation times. Real-time PCR was used to determine the mRNA level of ABCG2, BAX, and BCL2. The cellular uptake assay was performed to show the cellular uptake of nanoparticles. The size of NPs used in this study was about 179 nm with surface charge of −17.1 mV. MTT assay results showed that 1 μmol/L of free drug and 3 μmol/L of NPs could reduce HT29 cells by half (IC_50_) after 48 and 96 h, respectively. An increase in expression of BAX and a decrease in expression of ABCG2 were observed according to the real-time PCR. No significant change was detected in expression of BCL2. In conclusion, sufficient uptake of SN38-PEG-PLGA-Ver NPs and a significant decrease in expression of ABCG2 and an increase in expression of BAX and BAX/BCL2 ratio was observed after treatment with nanoparticles compared with free SN38. These results reveal that SN38-PEG-PLGA-Ver NPs can be an effective therapeutic method in colon cancer treatments and also may prevent anticancer drug resistance.

## Introduction

Colon cancer is the third leading cause of cancer-related mortality world wide, and the mortality from colorectal cancer has increased (Siegel et al. 2016). Chemotherapy is widely used to treat colorectal cancer; however, it might impose serious side effects (Candeil et al. 2004). Camptothecin analogues have been used for almost 20 years clinically to treat cancer. Irinotecan is used in combination with other chemotherapeutic drugs for metastatic colorectal cancer (Hagger and Boushey 2009). Camptothecin is a prodrug converted by carboxyl esterase into its active form, SN38. It has been established that SN38 exerted its cytotoxic activity through the inhibition of TOP1 (Gongora et al. 2011). SN38 interferes with topoisomerase I function by forming stable ternary complexes that prevent the dissociation of TOP 1. This ternary complex inhibits both replication and transcription and leads to formation of double strand DNA break and causes cell cycle arrest in S phase and apoptosis (Candeil et al. 2004). Unfortunately, limiting factors such as rapid rate of SN38 hydrolysis, cellular resistance to SN38, and the water insolubility of SN38 have limited the use of SN38 as an anti-tumor drug and increased the rate of treatment failure (Gottesman 2002). However, based on the literature, one of the more common causes of resistance to irinotecan and SN38 is the increased expression of ATP-binding cassette (ABC), subfamily G, isoform 2 protein (ABCG2), a drug efflux pump (Sukowati et al. 2012). This gene located in chromosome 4q22.1, is encoded as 655 amino acid proteins (Efferth 2015). Many in vitro studies have demonstrated that SN38 is a substrate for ABCG2, and high expression of ABCG2 is associated with decrease in drug potency (Nakanishi and Ross 2012).

Several studies have attempted to find a new method to overcome these side effects and improve anti-tumor efficacy of SN38, focusing on designing an efficient system delivery. It has been reported recently that the use of nanoparticles as drug delivery system can improve anticancer efficacy and overcome these side effects (Milane et al. 2011; Xue and Liang 2012; Gao et al. 2015).

Polylactic-*co*-glycolic acid (PLGA) is one of the most famous polymers due to its biodegradability and poor stability in water without any serious side effects. PLGA nanoparticles modified with polyethylene glycol (PEG) are stable in water (Locatelli and Franchini 2012; Zhang et al. 2014).

Programmed cell death or apoptosis can be induced through extrinsic and intrinsic pathways regulated by proteins. The intrinsic pathway is mediated by anti-apoptotic proteins such as BCL2 and proapoptotic protein Bax (Elmore 2007; Xu et al. 2013). It has been proposed that the expression ratio of BAX/BCL2 (proapoptotic to anti-apoptotic molecules) is related to the sensitivity of cells to apoptosis (Eimani et al. 2014). It has been concluded that the BCL2/BAX expression ratio may be considered as prognostic factor in low grade urinary bladder cancer (Gazzaniga et al. 1996), and also loss of BCL2 expression can be a prognostic factor in recurrent colon cancer (Poincloux et al. 2008).

The aim of this study is to demonstrate cytotoxic effect and activity of SN38-PEG-PLGA-verapamil nanoparticles on HT29 cancer cells and based on the above, we investigated the change in mRNA expression level of ABCG2 and BAX and BCL2 gene after treatment with SN38-PEG-PLGA-Ver nanoparticles. It is assumed that the PLGA nanoparticle modified with PEG can increase the stability of the nanoparticle and it is in this respect NPS may increase the cytotoxicity effect of drug and overcome the side effect of chemotherapy.

## Materials and methods

### Nanoparticle characterization

These nanoparticles consisted of biodegradable poly(lactic-*co*-glycolic acid)–poly(ethylene glycol)-verapamil-SN38. The nanoparticles were gifted by Faculty of Pharmacy at Tehran University of Medical Sciences. The surface morphology of nanoparticles was determined by scanning electron microscopy (Philips, model XL30-the Netherlands). The solution containing nanoparticles was dried on a cover slip and a thin coating of gold was expanded on it and then loaded in microscope.

Zeta potential, particle size and size distribution of nanoparticles were characterized by a light scattering instrument (ZetasizerNanoZS, Malvern, UK). To determine the size of NPs, 1 mL of the solution containing nanoparticles was loaded in instrument.

### Drug loading

Ten milligrams of NPs was suspended in 10 mL DMSO (Merck, Germany). To release drug from NPs, first 20 mL of methanol was added and then 60 mL of water was added to the suspension and 50 μL of the supernatant was analyzed with HPLC (Waters, Milford, USA) at a wavelength of 256 nm. Drug loading was measured by the mass ratio of the drug in nanoparticles to the weight of NPs.

### Cell culture

Human colon adenocarcinoma cell line HT29 was gifted by the national cell bank of Iran (NCBI). HT29 cell line was maintained in the DMEM medium (BioSera, England) with 10% fetal bovine serum (Gibco, USA) and 1% kanamycin (Sigma-Aldrich, USA) at 37c in 5% CO_2_ in humified atmosphere. Cell culture medium was changed every day. The cells were kept in exponential growth phase by continuous culture.

### In vitro cell viability measurement by MTT assay

Cell viability was determined by the 3-(4,5 dimethylthiazol-2-yl)-2,5-diphenyltetrazolium bromide (MTT) assay (Sigma-Aldrich, USA). In brief, HT29 cells with 80% confluence were washed with PBS and detached by trypsin/25% EDTA and they were seeded into 96-well plates at a density of 1 × 10^4^ cells/well and incubated for 24 h to reach approximately 80% confluence. Then, cells were treated with desired concentrations of SN38 (Knowshine, China) (1, 5, 10 and 20 μmol/L) and SN38-PEG-PLGA-Ver (Resomer^®^ RG 504 h Evonik Rohm-Germany) (0.5, 1, 3, 5, 7, 10, 15 and 20 μmol/L). Seven wells were included in each concentration. Untreated cells with culture medium were served as control. For providing SN38 drug solution, 0.5% DMSO and DMEM medium were used, and for providing SN38-PEG-PLGA-Ver nanoparticle solution, deionized water and DMEM medium were used. After incubation for 24, 48, 72 and 96 h, 100 μL media with 0.5 mg/mL of MTT solution was added to each well and incubated for 3–4 h. In the next step, the medium containing MTT was carefully removed and 150 μL of DMSO (dimethyl sulfoxide) was added into each well to dissolve the formazan crystal. The 96-well plates were gently shaken for 15 min at 37 °C. The absorbance was finally measured with a microplate reader (Bio-Rad, USA) at 570 with 630 nm as reference. The absorbance of untreated cells was considered as the indicator of 100% cell viability. The effect of free SN38 and SN38-PEG-PLGA-Ver on the viability of HT29 cells were expressed as the % viability, using the following formula: cell viability (%) = absorbance_560_ treated cells/control cells × 100. The IC_50_ values were defined as SN38 and SN38-PEG-PLGA-Ver concentrations that cause 50% reduction of untreated wells.

### DAPI staining (morphological assessment of apoptotic cells)

To identify apoptotic bodies, HT29 cells were implanted on cover slip in 6-well plate and incubated with or without SN38-PEG-PLGA-Ver (3 μmol/L) for 24 h and washed twice in PBS 1% for 10 min and fixed with formaldehyde 4% for 10 min and again washed in PBS 1% for 10 min. Next, the cells were treated with Triton-X 100 for 10 min and washed with PBS 1% for 10 min and stained with DAPI (1 μg/mL) for 5 min. After washing with PBS 1% for 10 min, the morphology of apoptotic cells was observed by fluorescence microscope (Bell, INV-100FL).

### Cellular uptake of nanoparticles (cellular uptake assay)

In this study, coumarin-6 was used as fluorescent molecule, which can be loaded in the PLGA nanoparticle to determine the cellular uptake by HT29 cancer cells. HT29 cells were implanted on cover slip in 6-well plates and incubated for 2 h with coumarin-6-loaded PEG-PLGA (3 μmol/L) and fixed with formaldehyde for 10 min, and they were stained with DAPI for 3–5 min to display the nuclei. After washing with PBS, the cells were observed by fluorescence microscopy. The images of the nuclei of the cells treated with coumarin-6-loaded-PEG-PLGA and stained with DAPI were taken with the following channels: DAPI (blue channel) at 340 nm and coumarin-6 (green channel) at 485 nm.

### Total RNA extraction and reverse transcription

The HT29 cells were seeded into each well of the 6-well plate in triplicate at a density of 1 × 10^5^. Then, the cells were cultured for 48 and 96 h treatment with their respective IC_50_ value of SN38 and SN38-PEG-PLGA-Ver NPs, for being used for the RNA extraction. Total RNA was extracted from HT29 cells using easy blue (Trizole) according to the protocol. For further analysis, the extracted RNA was dissolved in DEPC-treated water and stored at −80 °C. The RNA purity was determined by measuring the ratio *A*_260_/*A*_280_ (IMPLEN GmbH, Germany) with purity values of 1.8–2.0.

### cDNA synthesis

2 μg of total RNA was performed with Revert Aid First strand cDNA synthesis Kit (Fermentas, USA) according to the manufacturer’s protocol. 20 μL of reaction volume was performed with iCycler thermal cycler (Bio-Rad; Hercules, CA) at 25 °C for 5 min, 42 °C for 60 min and 70 °C for 5 min and the cDNA was stored at −20 °C until being used. The primers were designed with NCBI online tools. The integrity of cDNA was confirmed by PCR of the housekeeping gene, *β2M* (as an endogenous control gene), using specific primers (Table 1) according to the Taq DNA Polymerase 2× maser mix RED (Amplicon, Denmark). The PCR product was separated on a 2% agarose gel.

**Table 1:** List of primers

Gene	Forward	Reverse
*ABCG2*	*TCAGCTGGTTATCACTGTGAGG*	*GGCTCTATGATCTCTGTGGCT*
*BCL2*	*CGGAGGCTGGGATGCCTTTGT*	*CAAGCTCCCACCAGGGCCAAA*
*BAX*	*TGCCTCAGGATGCGTCCACCAA*	*CCCCAGTTGAAGTTGCCGTCAG*
*β2M*	*AGATGAGTATGCCTGCCGTG*	*GCGGCATCTTCAAACCTCCA*

### RT-qPCR

The quantitative reverse transcription PCR of ABCG2 and BAX and BCL2 was performed according to the SYBR green protocol (Amplicon, Denmark). 25 μL of reaction volume contains 20 ng of cDNA and 0.5 Μl of ABCG2 specific primers (Table 1) and 13 μL of Real Q Plus 2× Master Mix and 6 μL of PCR-grade H_2_O. Reactions including ABCG2 gene and *β2M* gene as reference genes were analyzed and run on a multi-color real-time PCR detection system (Bio-Rad, USA). Average Ct triplet repeats were used for data analysis. The cycling parameters were analyzed and determined using statistical analysis. The integrity of real-time qPCR products were confirmed by separating on a 2% agarose gel.

### Statistical analysis

Statistical analysis was performed with SPSS 21 software. The one-way ANOVA was used for the analysis of all MTT assay data. The P value was considered to be statistically significant. Cycling parameters of real-time qPCR were analyzed and determined using the Pfaffl modification, and gene expression data were normalized and compared with housekeeping *β2m* gene by LinReg software, and expression level of gene was calculated by REST program.

## Results

### Characterization of SN38-PEG-PLGA-Ver NPs

**Fig. 1:**
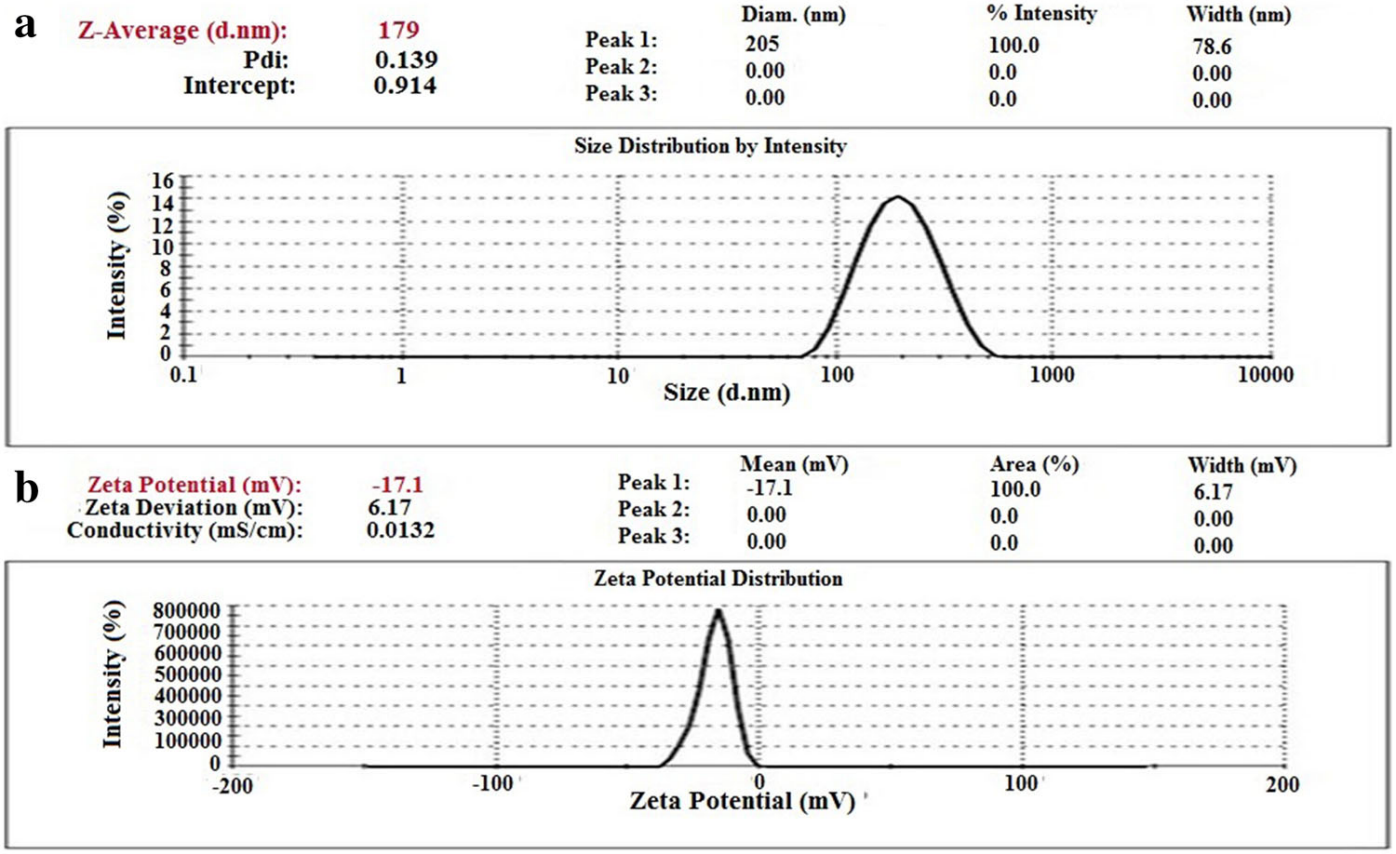
**a** Size and size distribution of NPs. **b** Zeta potential of NPs

The nanoparticles were gifted by the Faculty of Pharmacy at Tehran University of Medical Sciences. To determine the surface morphology of NPs, scanning electron microscope was used. The SN38-PEG-PLGA-Ver NPs had a pleasant spherical shape with clear and smooth surface and the size distribution of NPs was monodispersed (Fig. 1).

**Fig. 2:**
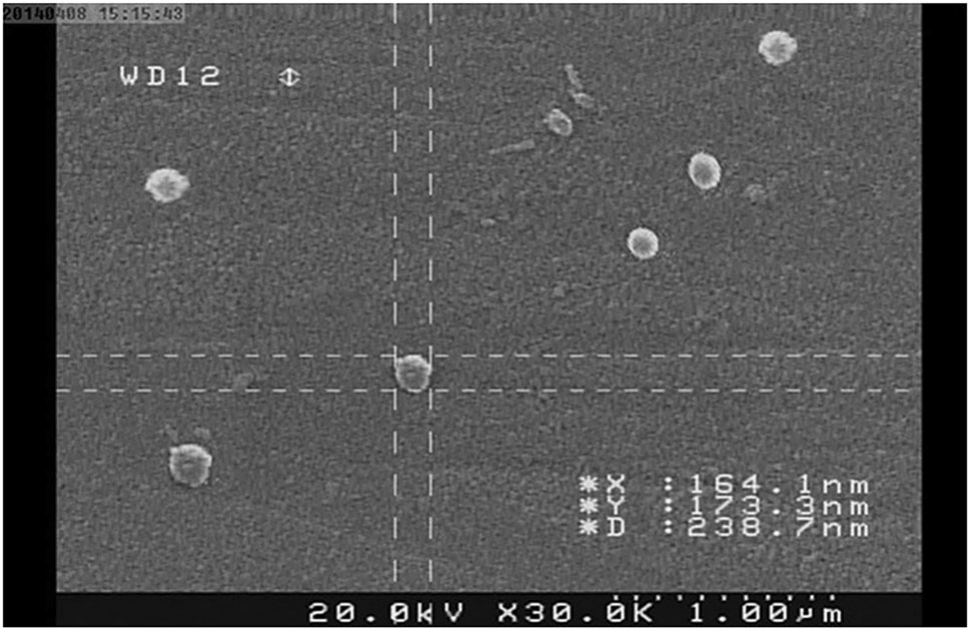
SEM micrographs of SN38-PEG-PLGA-Ver NPs showing the shape and surface

Zeta sizer was used to characterize the size of nanoparticles. The mean particle size of NPs was 179 nm and their polydispersity index, which indicates the size distribution, was 0.139. Zeta potential of NPs was −17.1 mV. The negative charge was because of the presence of carboxylic groups in PLGA. Finally, the drug loading was 6.03 (Fig. 2).

### MTT assay

**Fig. 3:**
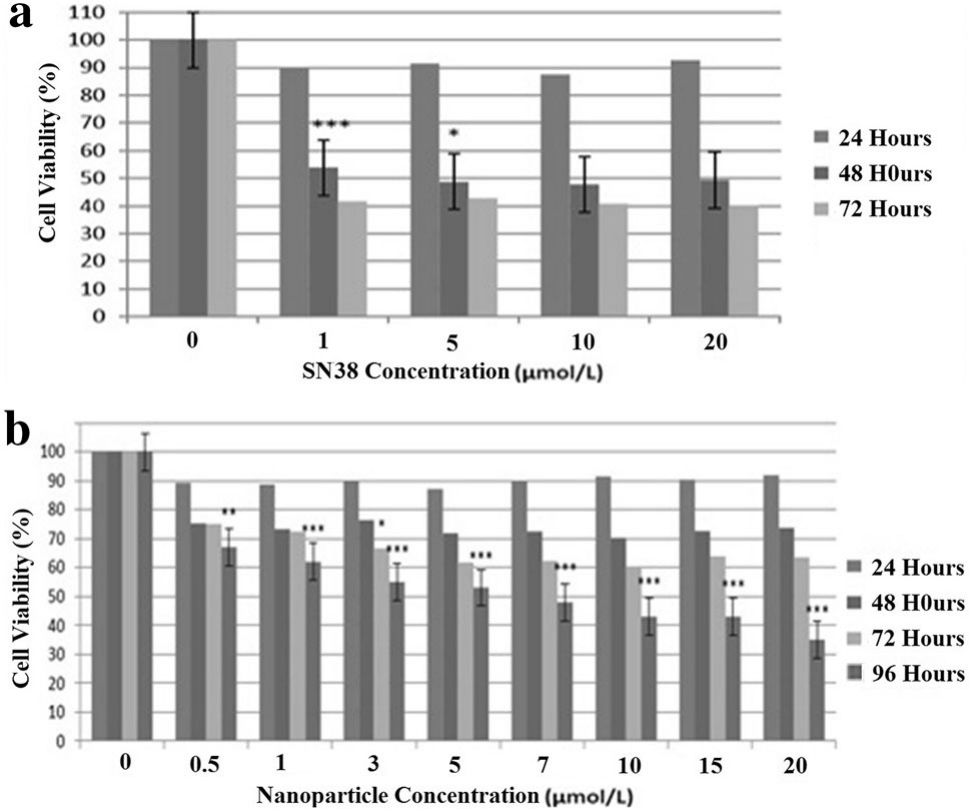
**a** The results of MTT assay after treatment of HT29 cell for 24 and 48 and 72 and 96 h with different concentrations of free SN38. **b** The results of MTT assay after treatment of HT29 cell for 24 and 48 and 72 and 96 h with different concentrations of SN38-PEG-PLGA-Ver nanoparticle (*p* values between 0.001 and 0.00 are shown with three asterisks, *p* values between 0.001 and 0.01 are shown with two asterisks and *p* values between 0.01 and 0.05 are shown with one asterisk)

To explore the cytotoxicity of free SN38 and SN38-PEG-PLGA-Ver NPs on colon cancer cells, the MTT assays were initially used. The results evidenced a significant reduction of cell viability in treated cells with nanoparticles compared with control cells (*p* < 0.05). As shown in Fig. 3, free SN38 and SN38-PEG-PLGA-Ver NPs showed a concentration-dependent cytotoxicity effect on HT29 cell line, and a significant reduction of cell viability after 48 h of treatment with free SN38 and after 96 h of treatment with SN38-PEG-PLGA-Ver was compared with control cells (*p* < 0.05). Free SN38 and SN38-PEG-PLGA-Ver NPs exerted cytotoxicity effect on HT29 cells with IC_50_’s value of 1 μmol/L for 48 h and IC_50_’s value of 3 μmol/L for 96 h, respectively. The IC_50_ values were used to measure and compare the effect of free SN38 and SN38-PEG-PLGA-Ver NPs on cell viability. According to the results, there is no significant difference between cytotoxicity effect of free SN38 and SN38-PEG-PLGA-Ver (*p* < 0.05).

### Morphological assessment of apoptotic cells

**Fig. 4:**
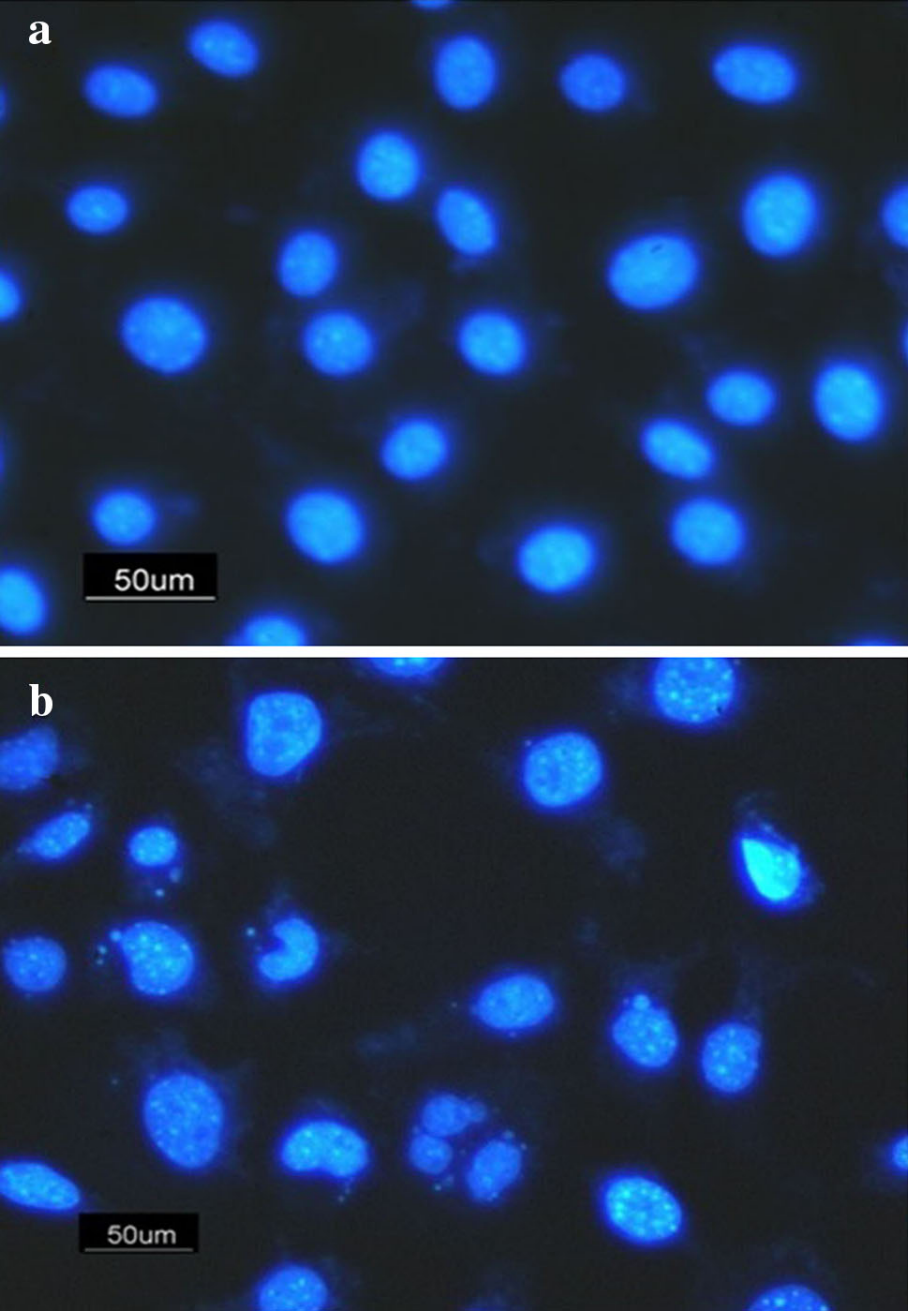
DAPI staining: **a** untreated HT29 cells as control, **b** HT29 cells treated with nanoparticle (3 μmol/L)

The therapeutic effects of the drug-loaded polymeric nanoparticles were dependent on the ability of induction apoptosis. To determine the changes in apoptotic cells treated with SN38-PEG-PLGA-Ver nanoparticles, HT29 cells were stained with DAPI to analyze the morphology of apoptotic cells. After 96 h of treatment with SN38-PEG-PLGA-Ver NPs (3 μmol/L) HT29 cells showed changes in their nuclei. As shown in Fig. 4, small holes appeared in the nuclei of treated cells, and DNA fragmentation was clearly evident. In comparison, untreated cells had normal nuclei and exhibited normal morphology.

### Cellular uptake assay

**Fig. 5:**
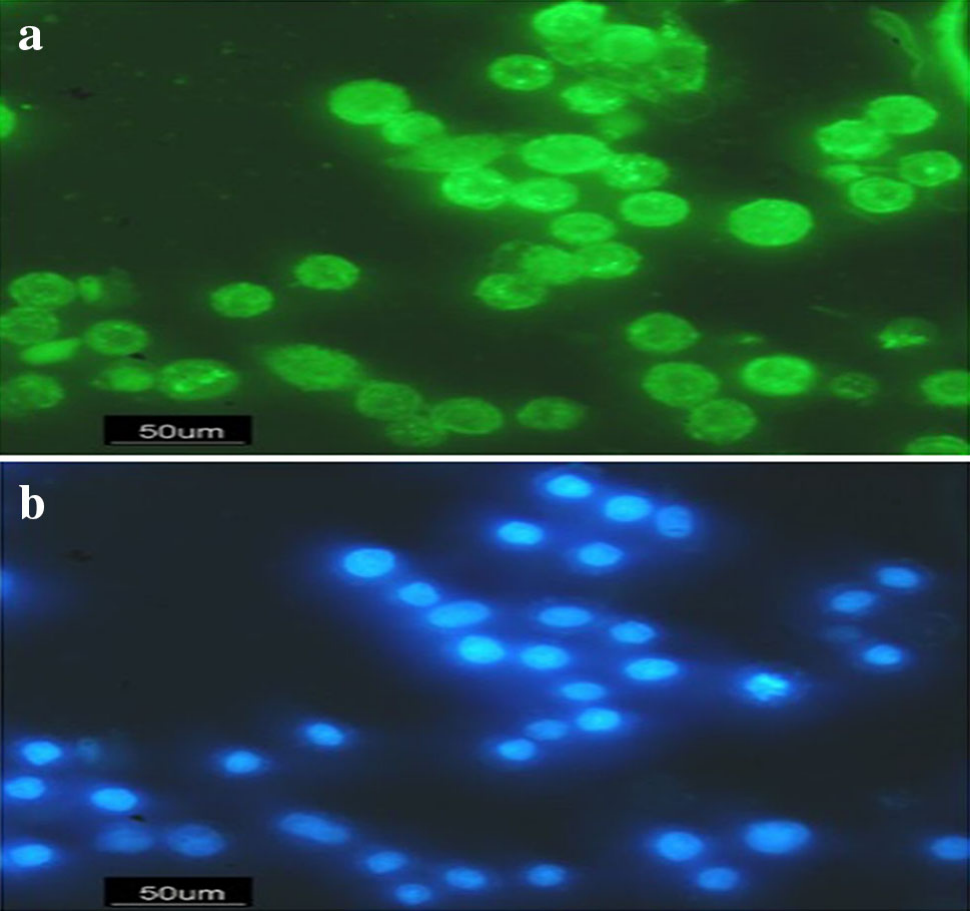
Cellular uptake: **a** HT29 cells treated with coumarin-6-loaded SN38-PEG-PLGA nanoparticles at 485 nm **b** HT29 cells treated with coumarin-6-loaded SN38-PEG-PLGA nanoparticles at 340 nm

The therapeutic effects of the drug-loaded polymeric nanoparticles were dependent on internalization and sustained retention of nanoparticles by tumor cells. The in vitro studies were capable of providing some circumstantial evidence to show the advantages of the nanoparticle formulation compared with the free drug. coumarin-6 served as a fluorescent probe in an attempt to represent the drug in the nanoparticles for visualization analysis of cellular uptake of the nanoparticle. Figure 5 shows the image of HT29 cells after 2 h of incubation with the coumarin-6-loaded PEG-PLGA nanoparticle at the concentration of 3 μmol/L. The image was taken with the following channels: DAPI (blue channel) at 340 nm and coumarin-6 (green channel) at 485 nm. The image shows that the coumarin-6-loaded PEG-PLGA nanoparticles (green) have entered into cytoplasm, and the blue nuclei stained by DAPI indicates that the coumarin-6-loaded PEG-PLGA nanoparticles are situated around the nuclei (blue, DAPI) and normal nuclei.

### Gene expression analysis

**Fig. 6:**
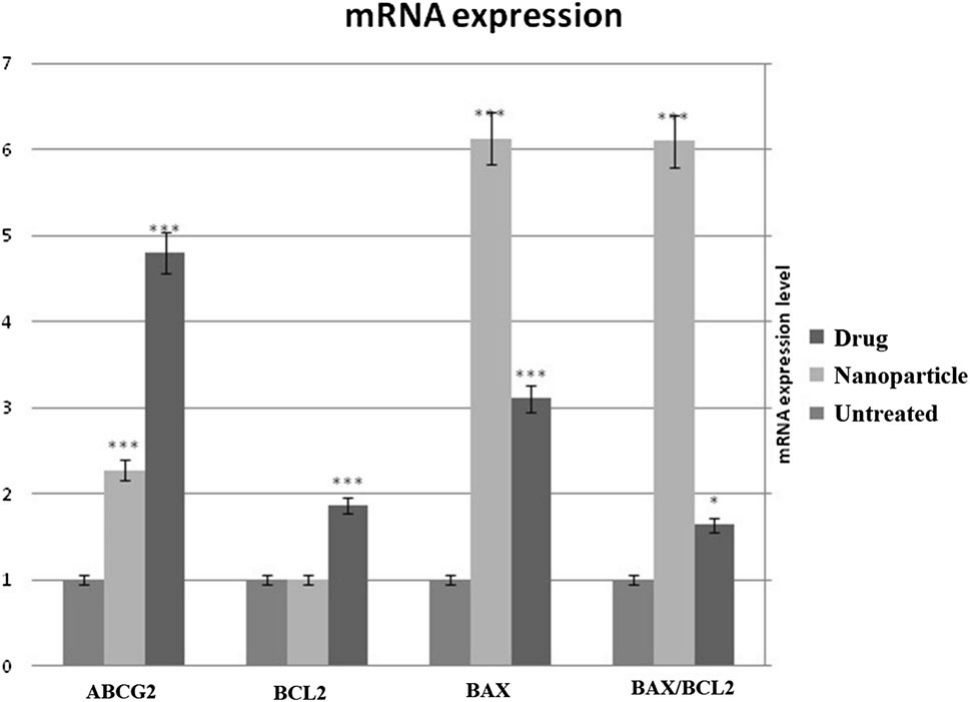
Real-time PCR results of expression level for *ABCG2*, *BAX* and *BCL2* genes in untreated cells, nanoparticle-treated cells and drug-treated cells nanoparticle. (*p* values between 0.001 and 0.00 are shown with three asterisks, *p* values between 0.001 and 0.01 are shown with two asterisks and *p* values between 0.01 and 0.05 are shown with one asterisk)

Several studies indicate that ABCG2 is potentially involved in cancer drug resistance and increased expression of ABCG2 can increase the risk of drug resistance (Efferth 2015). To investigate changes in ABCG2 expression following treatment, the level of ABCG2 mRNA expression of HT29 cancer cells treated with an IC_50_ dose of SN38 and SN38-PEG-PLGA-Ver nanoparticles were analyzed by quantitative real-time PCR using SYBR-Green. The untreated cells were defined as control (1.0). As can be seen in Fig. 6, after analyzing real-time PCR data by REST and SPSS software, the level of ABCG2 mRNA expression was significantly different compared with control sample, and it was 4.793-fold greater after treatment with IC_50_ of SN38 (1 μmol/L) (*p* = 0.00). In addition, the level of ABCG2 mRNA expression was significantly different compared with control sample, and it was 2.277-fold greater after treatment with IC_50_ of SN38-PEG-PLGA-Ver NPs (3 μmol/L) (*p* = 0.00). Furthermore, the difference between SN38-treated group and SN38-PEG-PLGA-Ve-treated group was statistically significant (*p* = 0.022).

According to the MTT assay and DAPI staining results, SN38-PEG-PLGA-Ver nanoparticles had cytotoxic effect on HT29 human colon cancer cells by inducing apoptosis. It has been reported in the literature that the estimation of BAX/BCL-2 expression ratio is one of the standard ways to determine the vulnerability of a cell to apoptosis (Eimani et al. 2014). To determine the vulnerability of HT29 cells after treatment with SN38-PEG-PLGA-Ver NPs in comparison to SN38 treatment, the level of BAX and BCL-2 mRNA expression were measured. The untreated cells were defined as controls (1.0). As shown in Fig. 6, after treatment with IC_50_ of SN38-PEG-PLGA-Ver, there was no significant difference in BCL-2 expression in comparison to untreated cells (*p* = 0.62). However, after treatment with IC_50_ of SN38, the level of BCL-2 expression was 1.883-fold greater (*p* < 0.05). Furthermore, the difference between SN38-treated group and SN38-PEG-PLGA-Ver-treated group was statistically significant (*p* < 0.05).

As can be seen in Fig. 6, after treatment with IC_50_ of SN38-PEG-PLGA-Ver NPs and with IC_50_ of SN38, the level of BAX expression was increased 6.1- and 3.1-fold, respectively, compared to control sample (*p* < 0.05). Moreover, the difference between SN38-treated group and SN38-PEG-PLGA-Ver-treated group was statistically significant (*p* < 0.05).

After treatment with IC_50_ dose of SN38 and SN38-PEG-PLGA-Ver nanoparticles, the BAX/BCL-2 expression ratios were increased 1.64- and 6.1-fold, respectively, and these differences were statistically significant (*p* < 0.05), as can be seen in Fig. 6. In addition, the difference between SN38-treated group and SN38-PEG-PLGA-Ver-treated group was statistically significant (*p* < 0.05).

## Discussion

SN38 is one of the chemotherapeutic agents widely used to treat colorectal cancer (Zhou et al. 2013). SN38 interferes with topoisomerase I function by forming stable ternary complexes that prevent the dissociation of TOP 1 and cause cell cycle arrest in S phase and apoptosis. It has been found that ABCG2 expression is associated with cancer resistance to SN38 and it has also been shown that ABCG2 over-expressed in colon cancer samples with SN38 resistance (Xie et al. 2014). In this study, SN38-PEG-PLGA-Ver nanoparticles were prepared in which PEG-PLGA was used as drug carrier. The size of SN38-PEG-PLGA-Ver nanoparticle was 179 nm which enabled them to escape from the immune system (opsonization). In addition, the acceptable small size of nanoparticle can increase blood circulating time of nanoparticles and cellular uptake. It has been shown that the PLGA nanoparticle modified with PEG can increase the stability of nanoparticle in water due to the hydrophilic and nonionic effect of PEG in water.

In this study, first, MTT assay was used to explore the anticancer effect of SN38-PEG-PLGA-Ver nanoparticles and free SN38. The results showed no cytotoxicity effect of free drug for 24 h. The reason for this is that drug needs more than 24 h to enter the cells and be effective. The results also demonstrated that free SN38 had more cytotoxicity effect with a gentler gradient after 72 h, which may be due to the rapid rate of free SN38 hydrolysis and ABCG2 activity pumping out free SN38, while SN38-PEG-PLGA-Ver had the most cytotoxicity effect at 96 h. The increase in the action time of nanoparticle was because of the time they need to enter cells and release the drug, and based on these results, it can be concluded that SN38-PEG-PLGA-Ver nanoparticles could increase stability of SN38 from 48 to 96 h and also increased solubility of SN38 in water, due to the water insolubility of SN38. In addition, increased stability of SN38 by nanoparticle may propose that SN38-PEG-PLGA-Ver nanoparticles entered the cells through endocytosis and delay SN38 hydrolysis and can escape from the ABCG2 and P-gp pumps which can lead to preventing and overcoming drug resistance. ABC family proteins can play an important role in cancer drug resistance due to extruding of drugs from the cells. Studies have been shown that ABCG2 over-expression represents a potential mechanism of resistance to SN38. In 2014, Sepehri et al. determined the cytotoxic effect of HAS-SN38 nanoparticles on HT29 cells and showed that HAS-SN38 nanoparticles cause an increase in SN38 stability and decrease side effects due to the targeted treatment (Sepehri et al. 2014). In 2015, Chen et al. showed that targeted delivery of 5-FU with EGF-grafted hollow mesoporous silica nanoparticle can overcome acquired drug resistance in drug resistance colorectal cancer cell line. They have determined that the mechanism of nanoparticles in overcoming drug resistance in SW480/ADR cells could be attributed to the increased stability of 5-FU and specific internalization of nanoparticles in colon cancer cells both of which can lead to higher intracellular drug accumulation (Chen et al. 2015). Furthermore, MTT assay results showed that IC_50_ value of nanoparticles was threefold higher compared with the free drug (3–1). This incompatibility was a result of partial release of SN38 from NPs. No significant difference was detected between cytotoxicity of the free drug and NPs which exhibited the cytotoxicity potential of NPs.

Besides, cellular uptake assay was used to determine the cellular uptake of nanoparticle. The results showed that free PEG-PLGA nanoparticles had sufficient cellular uptake which would be considered necessary for drug delivery systems. It has been reported in the literature that particle size plays an important role in the cellular uptake of biodegradable polymeric nanoparticle (Locatelli and Franchini 2012) and the appropriate size of PEG-PLGA nanoparticles improved the cellular uptake. In addition, DAPI staining showed that free PEG-PLGA NPs had no cytotoxic effect on cells because of the normal nuclei. In 2014, Koopaei shows that free PEG-PLGA nanoparticle has no effect on cell viability (Koopaei et al. 2014).

What we were interested in was whether nanoparticle SN38 can induce apoptosis and cause any change in nuclei appearance, DNA fragmentation, and BAX/BCL2 expression ratio; and whether it can alter ABCG2 expression to reduce SN38 resistance. DAPI staining performed to analyze morphological changes showed DNA fragmentation and an unusual change in nuclei of HT29 cells, an early apoptosis phenomenon probably, after treatment with IC_50_ value of SN38-PEG-PLGA-Ver nanoparticles. It has been proposed that the BAX/BCL2 expression ratio is related to the sensitivity of cells to apoptosis, and increasing the expression level of BAX and BCL2 indicate the induction of apoptosis (Raisova et al. 2001). Real-time PCR was used to determine changes in BAX and BCL2 expression induced by nanoparticle treatment. According to the results, the 1.64- and 6.1-fold change of BAX/BCL2 expression ratio after treatment with free drug and SN38-PEG-PLGA-Ver nanoparticles was a result of induction of apoptosis. In 2005, Souza et al. have found that BAX expression was upregulated after SN38 treatment in colon cancer cell line and no significant changes in BCL2 expression were detected (Souza et al. 2005).

After treatment with nanoparticles, there were no significant changes in BCL2 expression, and *BAX* expression increased 6.1-fold, and BAX/BCL2 expression ratio increased 6.1-fold compared with the reference gene. According to the results, it may be confirmed that SN38-PEG-PLGA-Ver nanoparticles induce apoptosis in a different way of *BCL2*. The significant increase in BAX/BCL2 expression ratio proved the induction of apoptosis, and morphological changes showed in DAPI staining.

According to the research done by Candeil, SN38 treatment significantly increased expression of ABCG2 (Candeil et al. 2004). In 2017, Jouan et al. showed that Verapamil do not effectively inhibit ABCG2 and addition of Verapamil resulted in no significant changes in ABCG2 expression level in human cell (Jouan et al. 2005; Zhang et al. 2005). In the present research, the results obtained from real-time PCR showed a lesser increase in the level of ABCG2 expression after treatment with nanoparticles, compared with the sample treated with free SN38. In 2009, An and Ongkeko showed that inhibition of ABCG2 expression can reverse cancer chemoresistance and the reduction of ABCG2 expression can delay cancer chemoresistance (An and Ongkeko 2009). A lesser increase in the level of ABCG2 expression and an increase in the level of BAX expression may reduce the side effect and delay the SN38 resistance.

## Conclusion

In conclusion, the present study has shown that SN38-PEG-PLGA-Ver nanoparticles had cytotoxic effect on HT29 cells in a dose- and time-dependent manner. In addition, an increase in the level of BAX/BCL2 expression ratio and a lesser increase in the level of ABCG2 expression have been demonstrated, which may indicate that SN38-PEG-PLGA-Ver nanoparticles can serve as candidates to delay SN38 resistance and improve the induction of apoptosis and a new method to treat colon cancer.
